# Improved reliability, accuracy and quality in automated NMR structure calculation with ARIA

**DOI:** 10.1007/s10858-015-9928-5

**Published:** 2015-04-11

**Authors:** Fabien Mareuil, Thérèse E. Malliavin, Michael Nilges, Benjamin Bardiaux

**Affiliations:** Unité de Bioinformatique Structurale, CNRS UMR 3528, Institut Pasteur, 25-28 rue du Dr Roux, 75724 Paris Cedex 15, France; Cellule d′Informatique pour la Biologie, Institut Pasteur, 25-28 rue du Dr Roux, 75724 Paris Cedex 15, France

**Keywords:** Nuclear magnetic resonance, Automated NOE assignment, Structure determination, ARIA, CASD–NMR

## Abstract

In biological NMR, assignment of NOE cross-peaks and calculation of atomic conformations are critical steps in the determination of reliable high-resolution structures. ARIA is an automated approach that performs NOE assignment and structure calculation in a concomitant manner in an iterative procedure. The log-harmonic shape for distance restraint potential and the Bayesian weighting of distance restraints, recently introduced in ARIA, were shown to significantly improve the quality and the accuracy of determined structures. In this paper, we propose two modifications of the ARIA protocol: (1) the softening of the force field together with adapted hydrogen radii, which is meaningful in the context of the log-harmonic potential with Bayesian weighting, (2) a procedure that automatically adjusts the violation tolerance used in the selection of active restraints, based on the fitting of the structure to the input data sets. The new ARIA protocols were fine-tuned on a set of eight protein targets from the CASD–NMR initiative. As a result, the convergence problems previously observed for some targets was resolved and the obtained structures exhibited better quality. In addition, the new ARIA protocols were applied for the structure calculation of ten new CASD–NMR targets in a blind fashion, i.e. without knowing the actual solution. Even though optimisation of parameters and pre-filtering of unrefined NOE peak lists were necessary for half of the targets, ARIA consistently and reliably determined very precise and highly accurate structures for all cases. In the context of integrative structural biology, an increasing number of experimental methods are used that produce distance data for the determination of 3D structures of macromolecules, stressing the importance of methods that successfully make use of ambiguous and noisy distance data.

**Electronic supplementary material:**

The online version of this article (doi:10.1007/s10858-015-9928-5) contains supplementary material, which is available to authorized users.

## Introduction

Distances or contacts are of increasing importance in the determination of three-dimensional (3D) structures of biological macromolecules or complexes. Nuclear Magnetic Resonance (NMR) plays a privileged role in structural biology due to its ability to measure many distance restraints and thus making it possible to determine high resolution 3D structures. It is at the same time a tool of choice for studying dynamics, flexibility and function (Markwick et al. 2008). During the last 25 years, NMR structure calculation has been the subject of many developments (Güntert 2009; Williamson and Craven 2009; Guerry and Herrmann 2011) and metrics to validate NMR-derived structures are increasingly reliable (Nabuurs et al. 2006; Rosato et al. 2013). In the mean time, methods for NMR structure determination can be extensively tested, as large databases of NMR structures and data sets are available (Nederveen et al. 2005; Doreleijers et al. 2009).

Experimentally determined distance information is often ambiguous, and usually the set of determined distances needs to be filtered for false positives. Also, data may be inconsistent for various reasons, and distances may be absent. Even NMR measures only few distances, compared to the number of degrees of freedom, and whether or not the calculated structure is unique has always been a concern.

The structure calculation is therefore usually based on the generation of multiple conformers, all performed with identical data and identical parameters, and the convergence of these independent calculations is taken as an ad hoc criterion to assess the uniqueness of the obtained solution.

Recently, a Bayesian approach has been proposed for NMR structure determination (Rieping et al. 2005). Here, a very large number of structures are generated by a Markov-chain sampling algorithm, with the aim to calculate a probability distribution of all parameters (not only the coordinates). While this approach provides statistically meaningful estimation of structural uncertainty it is computationally much more expensive. Some aspects of this approach were introduced (Nilges et al. 2008) into classical NMR structure determination: (1) log-harmonic potential (Rieping et al. 2005; Nilges et al. 2006), (2) Bayesian weighting of the distance restraints (Habeck et al. 2006), corresponding to the forward and error models to evaluate the discrepancy of a structure from the data. Iterative re-weighting of the data provides a shortcut relative to full sampling of the weight on the experimental data. The proposed approach was benchmarked (Bernard et al. 2011) on a set of more than 300 protein structures with calibrated, assigned and selected distance restraints, and was shown to improve the precision and the structure quality. We introduced these features in the ARIA (Ambiguous Restraints for Iterative Assignment) protocol (Rieping et al. 2007) and used them during the Critical Assessment of Automated Structure Determination of Proteins from NMR Data (CASD–NMR) www.wenmr.eu/wenmr/casd-nmr (Rosato et al. 2009). We then realised that convergence was not attained in some cases and there was still room for improvement of structure quality.

The present work shows that limited modifications of the force field together with the log-harmonic restraint potential and a novel semi-automated way to determine criteria for the rejection of restraints improves the structure quality. Nine protein structures were used for validating the force field and fine-tuning the violation rejection criteria, all having been used for benchmarking in CASD–NMR (Rosato et al. 2012). Furthermore, a new procedure for determining the violation tolerance was introduced and was shown to improve the structure convergence. This procedure, along with pre-filtering of unrefined spectral peak lists, was tested on a new set of protein structures in order to define default values insuring convergence in a wide range of conditions.

## Material and methods

### Protein targets and input data sets

The following protein targets, from the CASD–NMR 1 data set (Rosato et al. 2009, 2012), were used for testing the approaches proposed here: these targets are denoted Vpr247, NeR103A, CGR26A, CtR69A, ET109A_ox, ET109A_red, atc0905, HR5537A, PGR122A (Table 1). The experimental NMR data consisted of unassigned 3D NOESY peak lists and chemical shifts assignments prepared with NESG (North East Structural Genomics consortium) protocols (www.nesg.org) and provided by CASD–NMR. For targets ET109A_ox and ET109A_red, the available residual dipolar couplings (RDC) restraints were also used for structure calculation.

Additional rounds of calculation were performed for ten protein targets from the CASD–NMR 2 data set (Table 2). In this case, two types of unassigned NOE peak lists were provided for each protein: (1) “unrefined” and (2) “refined” peak lists. Refined peak lists were generated by experienced NESG operators and used to calculate the final reference structures deposited in the PDB. Unrefined peak lists contained cross-peaks automatically picked in a preliminary analysis stage of spectra. For seven targets from CASD–NMR 2 (HR2876B, HR2876C, HR5460A, HR6470A, OR135, OR36, YR313A), RDC restraints were also used for structure calculation. NOE data were complemented with restraints on $\phi$ and $\psi$ dihedral angles predicted from backbone chemical shifts by making use of TALOS+ (Shen et al. 2009). The structure quality scores were determined with the Molprobity (Davis et al. 2007), CING (Doreleijers et al. 2012) and PSVS (Bhattacharya et al. 2007) validation suites. Molecular figures were prepared with the PyMOL Molecular Graphics System (Schrödinger, LLC).

### Simulated annealing and ARIA iterative protocols

The standard iterative protocol was used with ARIA version 2.3 (Bardiaux et al. 2012). For CASD–NMR 1 targets, the following protocol was used. Nine iterations with 50 conformers were calculated. The 15 conformers with the lowest value of energy were analysed to filter the set of distance restraints for false positives and assign ambiguities. For the calculations, we used an in-house computer cluster operating under Linux, and the Grid ReNaBi GRISBI (Blanchet et al. 2006a, b), making use of a dedicated version of ARIA (Mareuil et al. 2011). The “geometric” force field PARALLHDG (version 5.3), developed for NMR structure calculations with CNS (Brünger et al. 1998) and ARIA (Linge and Nilges 1999; Linge et al. 2003) and based on the standard force field for X-ray crystal structure refinement (Engh and Huber 1991), and PROLSQ non-bonded parameters (Konnert and Hendrickson 1980), were used. The simulated annealing protocol was applied through the standard ARIA 4-phases procedure (Rieping et al. 2007). Water refinement (Linge et al. 2003) was carried out in a 9 Å layer of TIP3P (Jorgensen et al. 1983) water and using OPLS (Jorgensen et al. 1996) non-bonded parameters. Detailed parameters used for CASD–NMR 2 targets are listed in Supplementary Table S4.

### Force field modifications

Two modifications of the PARALLHDG force field with PROLSQ non-bonded parameters were tested. First, the force constants for bond angles and improper dihedral angles were decreased by a factor 10, changing from 500 to 50 kcal mol$^{-1}$ rad$^{-1}$. Second, the van der Waals radii of hydrogen atoms were specifically increased, for hydrogen-hydrogen interactions only, as described in Table 3. This modification was implemented with NBFIX statements in CNS and no other scaling of atomic radii for the repulsive non-bonded potential was applied. The former hydrogen radii were small to avoid steric clashes between aliphatic hydrogens and the extended atoms defined in PROLSQ. The new radii were chosen to be in close agreement with the hydrogen radii used by Molprobity (Word et al. 1999).

### ARIA structure calculations

For CASD–NMR 1 targets, five sets of simulations were performed. *FBHW* and FBHWs* used a flat-bottom harmonic wall energy potential (FBHW) for distance restraints. *LogH* used a log-harmonic distance restraint potential with Bayesian weighting of restraints (Nilges et al. 2008). *LogHs* used a log-harmonic potential and reduced force constant for angles. FBHWs* and LogHs* included all additional force field modifications described above (reduced force constant for angles and bigger hydrogen radii). For blind calculations on CASD–NMR 2 data set, the LogHs* set-up was used for all targets.

### Violation monitoring

As other programs (Güntert 2004), ARIA uses “consistent violations” to identify false positives. A restraint is violated if the distance found in the structure lies outside the bounds by more than the violation tolerance $t$. To identify restraints that are systematically violated, each of the $S$-lowest energy structures in the ensemble is analysed. The fraction $\hbox {f}_i$ of structures violating restraint $i$ is calculated as:

1 $$\begin{aligned} f_i = S^{-1} \sum _{j=1}^S max\left( \theta (L_i - d^{i}_{j} - t), \theta (d^{i}_{j} - U_i - t)\right) \end{aligned}$$

where $\hbox {d}^i_j$ denotes the effective distance for restraint $i$ found in the $j$-th structure, $S$ is the number of structures analysed, $\hbox {L}_i$ and $\hbox {U}_i$ denote the lower and upper bounds of the $i$-th restraint and $\theta$ is the Heaviside function. We classify a restraint as violated if $\hbox {f}_i$ exceeds a user-defined violation threshold, which is set to 0.5 by default. In ARIA, the distance violation tolerance $t$ is usually entered as input from the user for iterations 0 to 8. Default values of 1000.0, 5.0, 3.0, 1.0, 1.0, 1.0, 0.1, 0.1, 0.1 Å have been determined in the past as giving good convergence results (Linge et al. 2001).

In the new version of ARIA, we modify this purely user-defined tolerance and make it follow the convergence of the calculation. For each restraint, the effective distance $d^i_{\rm eff}$ is calculated from the $S$-best-energy NMR conformers in an iteration, as the average sum of the inverse sixth power of the distance $d^{a,i}$ of each contributing assignment possibility $a$:

2 $$\begin{aligned} d^i_{\rm eff} = S^{-1}\sum _j^{S}\left( \sum _a {d^{a,i}_j}^{-6}\right) ^{-\frac{1}{6}} \end{aligned}$$

Each effective distance is compared to the target distance in the restraint list:

3 $$\begin{aligned} ec_i = d^i_{\rm eff} - d^i_{\rm target} \end{aligned}$$

From the list of differences $ec_i$ between effective and target distances, we obtain the standard deviation:

4 $$\begin{aligned} D = \sqrt{\langle ec_i^2\rangle - \langle ec_i \rangle ^2} \end{aligned}$$

where $\langle \ \rangle$ stands for averaging over the restraint list. The standard deviation $D$ is then multiplied by a parameter $T$ chosen by the user to produce the tolerance value $t$ used for rejecting violated restraints:

5 $$\begin{aligned} t = D T \end{aligned}$$

### Peak list pre-filtering for problematic CASD–NMR 2 targets

ARIA provides simple filtering of the input NOE peak lists which consists in discarding peaks for which no assignment possibility could be found on the basis of the chemical shift assignment lists and a tolerance window. We introduced two new types of pre-filtering to discard (1) weak cross-peaks and (2) potential artifactual cross-peaks from solvent. These two types of pre-filtering were applied only for the re-calculation of two problematic CASD–NMR 2 targets from unrefined peak list.

#### Weak NOE cross-peaks filtering

An NOE cross-peak $p$ is considered as weak if $I_{p}^{-1/6} > I_{min}^{-1/6} * 0.9$, where $I_{p}$ is the cross-peak intensity and $I_{min}$ is the smallest intensity found in the peak list. In other words, a weak cross-peak would give rise to a calibrated target distance longer that 90 % of the longest distance. Weak peaks are removed from the peak list by this filtering.

#### High density lines filtering

Strong solvent signals generate artifactual peaks that saturate the spectrum around the solvent resonances. Despite water suppression techniques, experiments ran in $\hbox {D}_2$O and awareness of peak-picking procedures, especially for water signal, the presence of solvent peaks in the peak list can hamper the assignment procedure. To discard cross-peaks that may correspond to solvent signals, we filter out high density lines in the ${}^1\hbox {H}$–${}^1\hbox {H}$ planes of a 3D NOESY peak list using the following procedure. First, we project all peaks on the HMQC or HSQC 2D plane. Second, we apply a grid on the 2D spectral plane using, along each of the spectral dimensions, a grid size corresponding to twice the assignment tolerance for the corresponding ${}^{13}\hbox {C}, {}^{15}\hbox {N}$ or ${}^1\hbox {H}$ nucleus. Third, the density of peaks $\rho$ (number of peaks) on each grid cell is computed as well as the average $\langle \rho \rangle$ and standard deviation $\sigma _{\rho }$ of the density over the full spectrum. Grid cells where $\rho > \langle \rho \rangle + n\sigma _{\rho }$ are then considered as high density lines in NOE planes and all cross-peaks within those cells are removed from the peak list. We tested values of 1, 2 and 3 for $n$ and observed that using $n=1$ gives the best selectivity owing to the fraction of filtered peaks from unrefined peak lists having a match in the corresponding refined peak lists (see Supplementary Table S3).

## Results and discussion

**Fig. 1:**
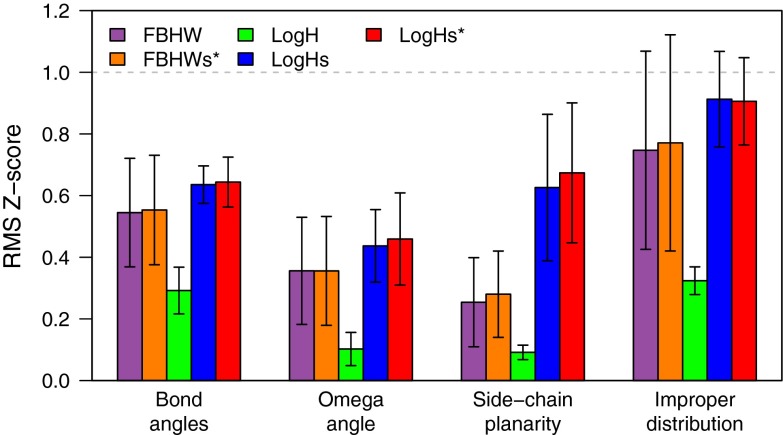
Average WHAT-IF RMS Z-scores according to the distance potential and the force field parameters used for CASD–NMR 1 targets calculated with ARIA. The WHAT-IF RMS Z-score of bond-angles, peptide-bond dihedral angles, side-chains planarity and improper dihedral angles are reported (average and standard deviation among all conformers calculated for CASD–NMR 1 targets)

The log-harmonic distance restraint potential has several key differences from flat-bottom harmonic wall (FBHW) potential. First, the target distance is a unique value, not a distance interval. Second, the log-harmonic potential increases sharply for distance values smaller than the distance target and is there more repulsive than the FBHW potential. Third, the log-harmonic potential is less attractive than the FBHW potential for distance values about three times the target distance value. These properties of the log-harmonic potential can in some cases lead to problems in convergence. These convergence issues are principally a consequence of restraint being less attractive than a harmonic or even than a linear restraint. This problem is exacerbated by the automated weighting procedure, which reduces the weight if the distance restraints are not well satisfied. This has the advantage that convergence is not “forced” by an incorrect distance restraint, but it also changes the balance between the contributions of the force field and the data in the energy function. For instance, we observed for CASD–NMR 1 targets, that structures calculated with the log-harmonic potential (*LogH*) display lower RMS Z-scores for local geometric parameters than the ones calculated with the FBHW potential (Fig. 1). Low RMS Z-scores, reported by WHAT-IF (Vriend 1990), reflect a too small number of outliers for the analysed parameters with regard to the distribution in high-resolution X-ray structures (Spronk et al. 2004).

The introduction of the log-harmonic restraint for distances changes the “philosophy” in the structure calculation from searching for geometrical consistency (the properties of the distance geometry algorithms used in the early days of NMR structure determination were the primary reason for the introduction of bounds) to searching for structures that present a compromise between having favourable “physical energy” (the force field) and satisfying the experimental data. Over-fitting is avoided by the automated weight determination, and distortions in the structure much less likely than for the standard flat-bottom potential, due to the resulting low weights on the distance restraints, and to the fact that the asymptotic slope of the logarithmic potential is zero. The two modifications that we propose in the PARALLHDG force field (Linge and Nilges 1999; Linge et al. 2003) take this into account. We soften the bond angle and improper dihedral angle terms in the force field, and we increase the size of the hydrogens to realistic values. We stress that both modifications only make sense together with the use of the log-harmonic potential for distance restraints, which replaces a purely geometric criterion by an energetic criterion, and makes relative weighting of experimental data and force field meaningful. The new, larger hydrogen radii introduce geometric inconsistencies that would make a distance geometry program abort during the “bound smoothing” phase.

Since the log-harmonic potential does not have bounds, the principal role of the bounds in ARIA is to decide which restraint is violated, and thus to select the peaks that are used for the structure determination. The standard procedure is purely user determined. The log-harmonic potential together with the automated weighting allows us to develop a statistically more meaningful criterion, which takes into account, in an iterative way, the convergence of the structure ensemble in each ARIA iteration to the experimental distance restraints (see “Material and methods” section).

### Impact of force field tuning

**Fig. 2:**
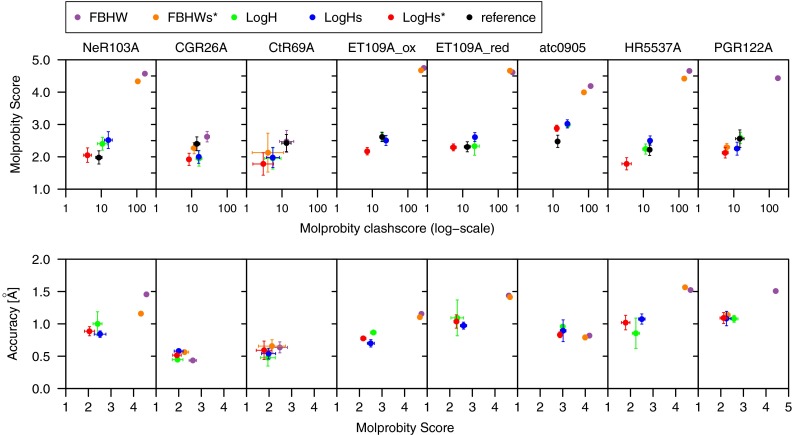
Quality scores according to the distance potential and the force field parameters used for CASD–NMR 1 targets calculated with ARIA. (*Top*) Molprobity Score versus molprobity clashscore in log-scale. Reference denotes the corresponding structure deposited in the PDB. (*Bottom*) Accuracy versus molprobity quality score

**Fig. 3:**
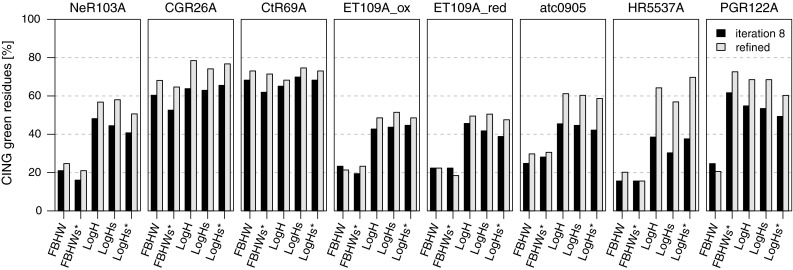
Percentages of green residues determined using CING ROG score on the conformations obtained in the last iteration and after water refinement for CASD–NMR 1 targets calculated with ARIA

The force field modifications introduced in “Material and methods” section were tested on a set of eight protein targets originating from the CASD–NMR initiative (Table 1) (Rosato et al. 2009). The quality of the NMR structures calculated by ARIA was analysed with the Molprobity clashscore and quality score (Fig. 2) as well as the CING ROG score (Fig. 3). A general trend of the calculations is the correlation between the improvements of structure convergence and quality. In all cases (except CtR69A and CGR26A), the number of clashes (clashscore) is drastically reduced by the use of the log-harmonic potential (*logH*) in comparison to the standard potential (*FBHW*). The introduction of bigger hydrogen radii improves the clashscore even further, but only in combination with log-harmonic potential (LogHs*), except for target PGR122A where FBHWs* also improves with regard to *FBHW*. The log-harmonic potential combined with softer force field and bigger hydrogen radii consistently gives the best Molprobity quality scores and clashscores, and is always better or similar to the scores of the reference PDB structures.

The good convergence and accuracy of the protein conformations generated by ARIA, expressed by the coordinate RMSD with the PDB structure are shown as a function of the Molprobity quality score (Fig. 2). The use of the log-harmonic potential (*LogH*) and its association with force field softening (*LogHs* and LogHs*) improves the Molprobity score and, to a lesser extend, the accuracy. For three targets (NeR103A, Et109A_red and HR5537A), the LogHs* calculations improved the ensemble precision compared to the *LogHs* calculations. The structure quality was also analysed by using the percentages of residues classified as green by CING (Fig. 3) as a criterion. For six targets, the percentage of green residues obtained with the standard potential (*FBHW*) does not exceed 25 %, even after water refinement. The use of the log-harmonic potential (*LogH*) as well as the softening of the force field (*LogHs* and LogHs*) consistently increase the percentage of green residues to the 40–60 % range. This percentage systematically improves after water refinement with the log-harmonic potential, whereas it is less beneficial with the standard bound-based potential (*FBHW*). The force field modifications in presence of the FBHW potential (FBHWs*) yields equal or worse CING scores than FBHW alone, except for target PGR122A.

We also validated the impact of the force field softening on the local geometry of structures calculated with ARIA. RMS Z-scores for bond angle, peptide bond torsion angle, side-chain planarity and improper angle distributions, calculated with WHAT-IF, were compared for the five different ARIA calculation set-ups (Fig. 1). As expected, the reduction of the force constant on bond angles and improper dihedral angles produces structures with better RMS Z-scores. While this improvement is not significant for the flat-bottom potential (FBHWs* vs. *FBHW*), it is remarkable when the log-harmonic potential is used (*LogHs*/LogHs* vs. *LogH*).

### Effect of adaptive violation tolerance on convergence and accuracy

The iterative generation of protein conformations based on NMR distance restraints, as implemented in the software packages ARIA (Rieping et al. 2007) and CYANA (Güntert 2004), uses a restraints list generated from the data before the actual structure calculation. At every iteration, the restraints are analysed and the most violated ones are removed from the list. The restraint rejection is a crucial step of the iterative calculation, as convergence can be missed because of a too large rejection rate, whereas a too low rejection rate will produce a set of inconsistent restraints which impairs also the convergence. To identify wrong assignments and noise peaks, the obtained restraints are subject to a violation analysis. In ARIA and CYANA, violation analysis relies on the hypothesis of structural consistency (Mumenthaler and Braun 1995). To assess whether a restraint follows the general trend imposed on the structures by the entire data set, we compare its distance bounds with the corresponding averaged distances observed in the ensemble of conformations (see “Material and methods” section: Eq. 2). The cutoff distance for considering a restraint as violated (violation tolerance) is reduced over iterations. The actual values were determined ad hoc to work well with the flat-bottom potential and have no statistical justification. With some of the CASD-NMR targets, we observed a convergence problem when using the log-harmonic potential regardless of the force field parameters. For instance, the VpR247 target (CASD–NMR 1) converges with the standard force field and the FBHW potential but not with the log-harmonic potential.

We propose to change this criterion to remove some of the arbitrariness and to be more consistent with the iterative determination of other parameters in ARIA, and the properties of the log-harmonic potential. The new violation tolerance in every iteration depends on the quadratic mean difference between conformers and target distance values (see “Material and methods” section: Eq. 5), in such a way that the effective tolerance is a consequence of the satisfaction of distance restraints in the previous iteration. The general idea guiding this procedure of adaptive parametrisation is to adjust the violation tolerance automatically to the quality of the experimental data. To estimate the data quality, we calculate the standard deviation of the differences between the effective and target distances. This number is used to scale the violation tolerance and hence allows the calculation to adapt the rejection level of the restraints to the fit of the obtained structures to the restraints. The worse is the fit, the larger is the standard deviation of the differences, and the more tolerant is the rejection of violated restraints.

**Fig. 4:**
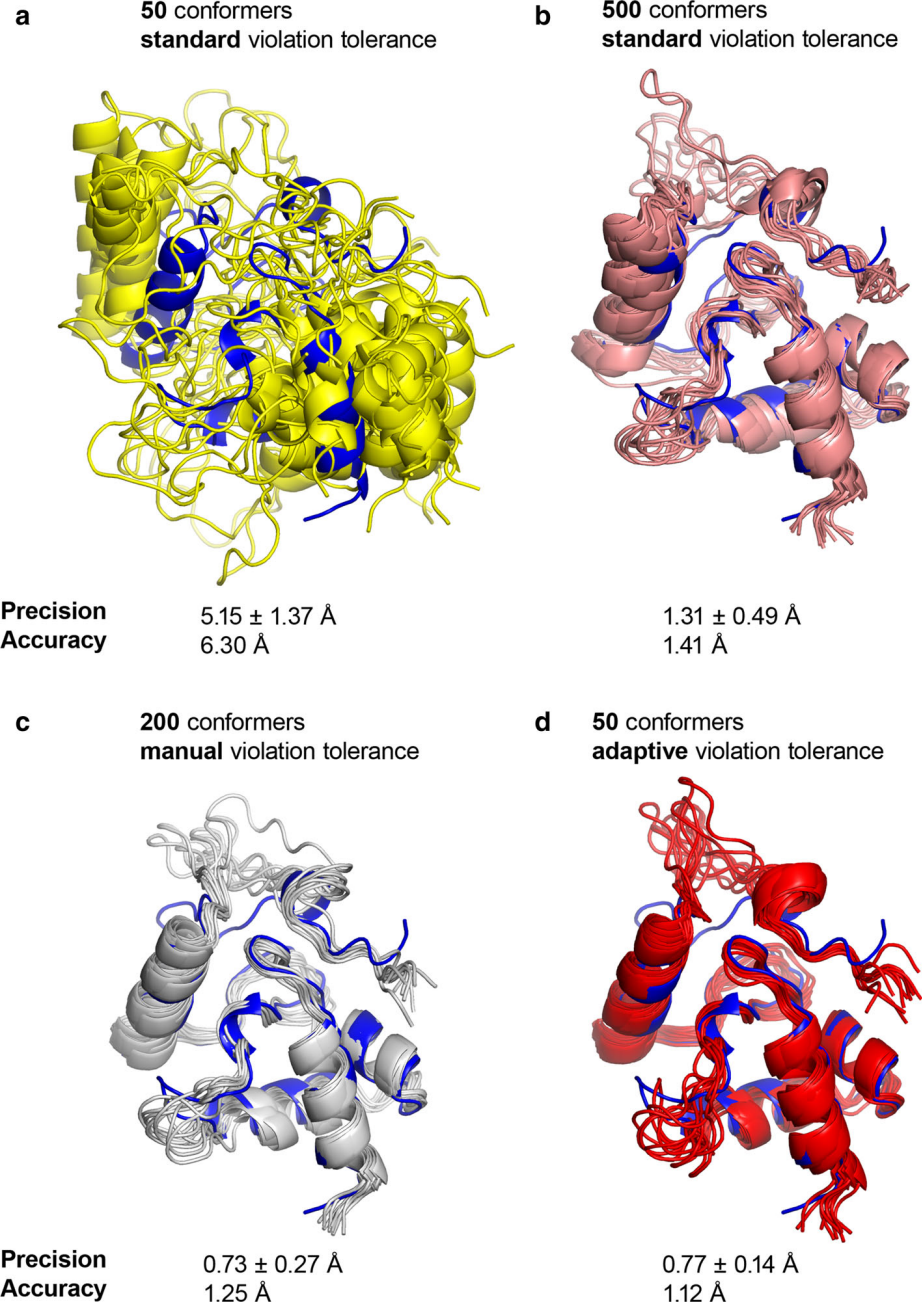
Conformers ensemble determined by ARIA according to the method used to determine the restraint violation tolerance for the CASD–NMR 1 target Vpr247. The average structure of the reference PDB entry is showed in *blue*. **a** Standard tolerance and 50 conformers per iteration. **b** Standard tolerance and 500 conformers per iteration. **c** Manual monitoring of the tolerance and 200 conformers per iteration. **d** Adaptive tolerance and 50 conformers per iteration

In order to set up a robust approach, we have derived a set of default values which allowed us to obtain convergence in most of the cases. For that purpose, two proteins (VpR247 and atc0905) were chosen, which are targets of the CASD–NMR 1 data set (Rosato et al. 2012) and that display opposite trends in convergence. VpR247 did not converge with the log-harmonic potential, whereas atc0905 converged with the standard violation tolerance variation. Extensive ARIA calculations were performed with the adaptive choice of violation tolerance, in order to analyse which sets of values result in convergence for VpR247, without hampering convergence for atc0905 . The final default values for $T$ are: 200, 6.0, 3.0, 2.0, 1.0, 1.0, 0.5, 0.5, 0.5 for iterations 0 to 8. Several ARIA calculations were performed on VpR247 and atc0905 with different parameters (Table 4). The convergence of the target VpR247 calculated with the log-harmonic potential is illustrated in Fig. 4. If the violation tolerance is set with the standard approach, one needs to calculate 500 conformations per iteration in order to obtain convergence (1.31 Å around average), and the calculation does not converge when 50 conformations are generated. If the tolerance is adaptively monitored as described above (see “Material and methods” section: Eq. 4), the convergence is obtained with 50 conformers per iteration, and the ensemble precision is improved to 0.77 Å. Furthermore, the obtained structure moves closer to the corresponding reference PDB structure, as the RMSD decreases from 1.41 down to 1.12 Å. Concerning atc0905, the convergence is observed in all cases with similar backbone accuracy, and the use of adaptive tolerance improves the backbone precision.

### ARIA blind calculations on CASD–NMR 2 data set

**Fig. 5:**
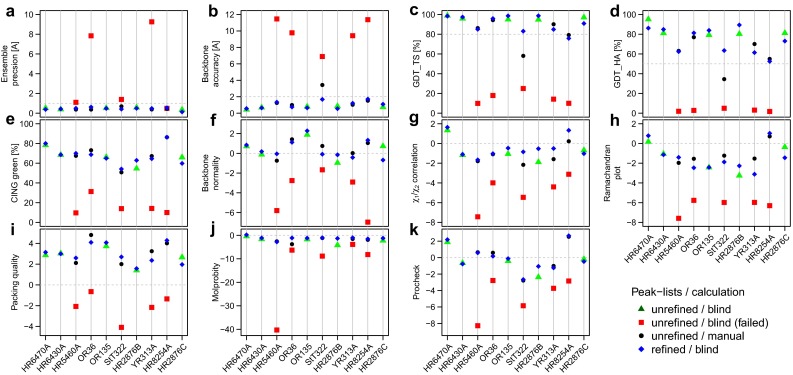
Average validation scores of the structures determined by ARIA on CASD–NMR 2 targets. Blind calculation starting from unrefined peak lists are represented as *triangle* (successful) or *square* (unsuccessful) while blind calculation starting from refined peak lists are represented as *diamond-shape*. Structures re-calculated from unrefined peak lists using manually optimised parameters are shown as *dot*. **a** Ensemble precision (average backbone RMSD between the conformers and the average conformer). **b** Ensemble accuracy (backbone RMSD between the average conformer and the average reference PDB structure). **c** Average GDT_TS (Global distance test, total score) between the average conformer and the average reference PDB structure. **d** Average GDT_HA (Global distance test, high accuracy) between the average conformer and the average reference PDB structure. **e** CING percentage of green residues. **f** Average backbone normality Z-score reported by WHAT-IF. **g** Average $\chi _{1}$/$\chi _{2}$ correlation Z-score reported by WHAT-IF. **h** Average Ramachandran plot appearance Z-score reported by WHAT-IF. **i** Average 2nd generation packing quality Z-score reported by WHAT-IF. **j** Average Molprobity clashscore Z-score reported by PSVS. **k** Average Procheck Z-score reported by PSVS

**Fig. 6:**
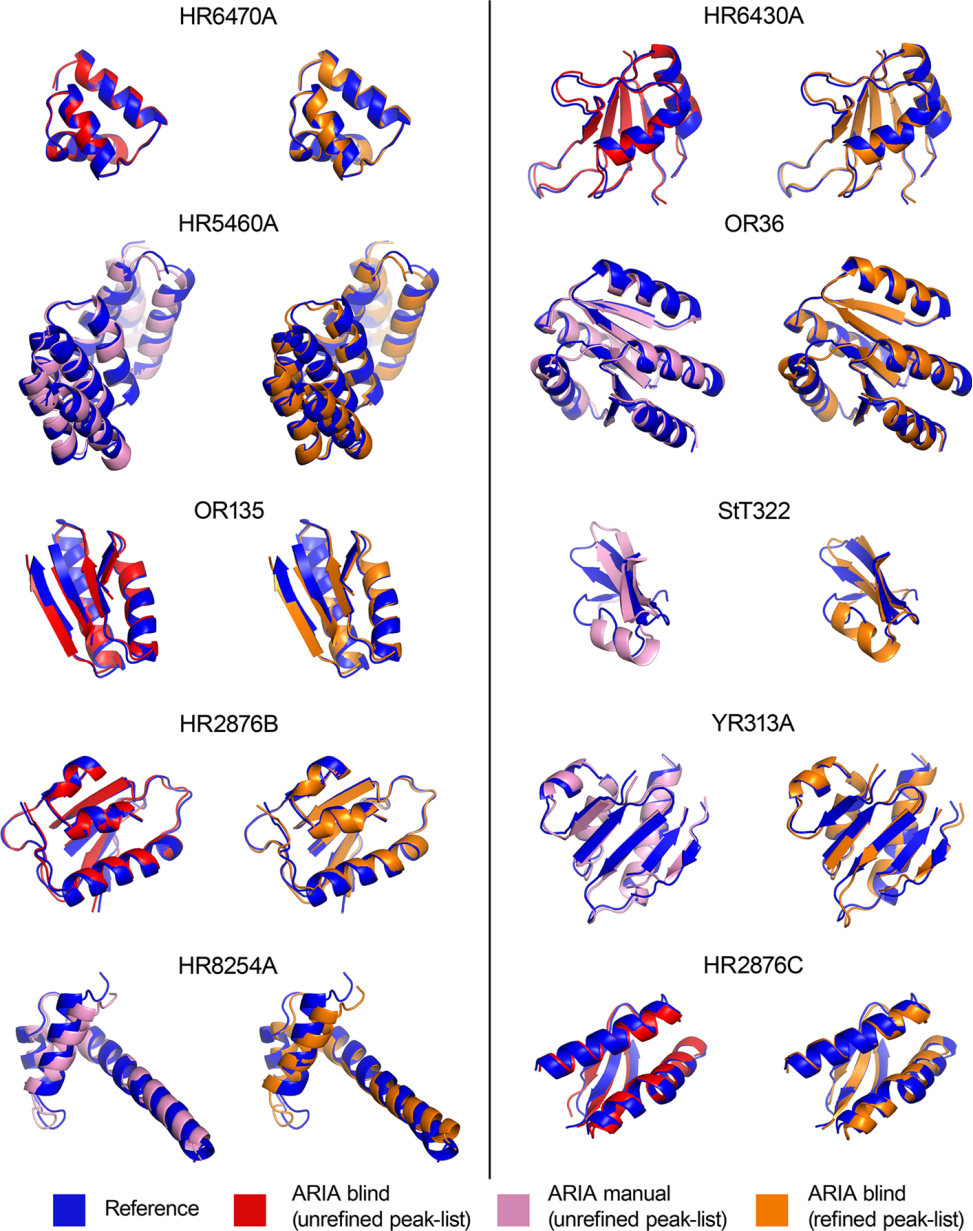
Overview of structures obtained with ARIA calculations for the ten targets from the CASD–NMR 2 data set. For each target, the average ARIA conformers in overlaid with the average reference PDB structure (in *blue*). Structures obtained by blind calculation from unrefined and refined peak list are shown in *red* and in *orange*, respectively. Structures re-calculated from unrefined peak lists using manually optimised parameters are shown in *pink*. Only the regions corresponding to ordered residues, determined by PSVS on the reference PDB structures, are drawn

While the primary purpose of the CASD–NMR initiative is to assess the reliability of automated approaches for NMR structure determination, it is also an invaluable resource of data for method development. The work presented above took advantage of the data from CASD–NMR 1 and our experiences with ARIA to validate approaches that we introduced as a consequence of our fully Bayesian approaches. CASD–NMR 2 served to evaluate the efficiency of ARIA with the improvements in automatically determining accurate NMR structures without knowing the actual solution. The sequence and NMR data (NOE peak lists and chemical shifts assignment) for ten targets (CASD–NMR 2) were provided prior to deposition of the final structures to the PDB. We ran ARIA calculations for the ten new targets using the improved setup that we had validated on CASD–NMR 1 targets: (1) log-harmonic potential with soften force field and bigger hydrogen atoms and (2) adaptive violation tolerance. At this stage, the new peak list pre-filtering functions were not used. Other parameters are listed in supplementary table S4. For each target, 3D NOESY peak lists were available in two flavours, corresponding to early (*unrefined*) and final (*refined*) stages of spectral analysis. In both cases, structure ensembles calculated with ARIA were submitted to the evaluators before public release of the final reference structure. Structure ensembles were analysed with the CING (Doreleijers et al. 2012) and PSVS (Bhattacharya et al. 2007) validation suites and the average scores are shown on Fig. 5 (see Supplementary Tables S1 and S2 for raw values). Structures determined by ARIA on the CASD–NMR 2 targets from *unrefined* and *refined* peak lists are shown in Fig. 6.

When refined peak lists were used, ARIA managed to determine well converged ensembles (RMSD $<$ 1 Å) for all ten targets (Fig. 5). In addition, ARIA ensembles are consistently very similar to the reference PDB structures. The mean backbone accuracy over ten targets is 1.1 $\pm$ 0.4 Å. The high accuracy is also reflected by the Global Distance Test (GDT (Zemla 2003)) results of ARIA ensembles. The GDT_TS (total score) is almost systematically greater than 80 % (except for target HR2854A with a GDT_TS of 76 %) and the high-accuracy score (GDT_HA) is always greater than 60 %. According to the criterion used in the original CASD–NMR 1 evaluation (Rosato et al. 2012), RMSD $<$ 2.0 Å or GDT_TS $\ge$ 80 %, ARIA calculations were successful in automatically determining accurate NMR structures for the ten targets. Structural quality of ARIA ensembles produced from refined peak lists is also very satisfactory. The percentages of green residues, determined by CING, range from 54 to 86 %. In addition, WHAT-IF Z-scores for backbone normality and $\chi 1$/$\chi 2$ angles correlation are constantly in the accepted range ($-2, +2$), and the average Molprobity clashscore Z-score over all targets is $-1.2\,\pm \,0.7$.

For structure calculations performed with ARIA using less optimised NOE data (unrefined peak lists), convergence is achieved for eight targets. For these proteins, ensemble RMSDs are smaller than 1 Å whereas for the unconverged targets YR313A and OR36, the ensemble precisions are 9.2 and 7.8 Å, respectively. Among the eight converged targets, three had a percentage of green residues less than 20 % (targets HR8254A, StT322 and HR5460A). On the basis of these two criteria, we considered that the structures generated by ARIA for targets YR313A, OR36, HR8254A, StT322 and HR5460A were not reliable and we did not submit them for further evaluation. It was later confirmed that the ARIA structures for these five targets were not accurate (RMSD from the reference PDB structures $>$7 Å). For the five other converged targets for which we submitted a structure ensemble, the successfulness criterion was achieved with an average accuracy $<$1 Å and a GDT_TS score $>$90 %. Moreover, the structural quality is comparable to what has been observed for ARIA structures calculated from refined peak lists.

CING ROG score revealed itself an excellent criterion for an objective detection of problematic or unsuccessful ARIA calculations. To give more rationality for the basis of this choice, we computed pairwise correlations between the different validation scores (Supplementary Figure S1) for ARIA calculations performed with refined and unrefined peaks list for all ten targets. The percentages of green residues correlates best with the ensemble accuracy (correlation coefficient of 0.91). In a sense, the CING ROG score can be considered as a consensus score of several scores from other validation tools such as WHAT-IF and PROCHECK (Laskowski et al. 1993) in addition to its own measures of quality. As expected, the ROG score correlates very well with these related scores (Supplementary Figure S1). Nevertheless, it performs better than any other individual score in detecting inaccurate solutions. We also observed that a threshold of 40 % of green residues is sufficient to discriminate between accurate and inaccurate structures.

Overall, we observed a noticeable improvement of the success rate of ARIA since the last evaluation round of blind calculations in CASD–NMR 1 (Rosato et al. 2012) where ARIA managed to get accurate solutions for only 75 % of the cases. Here, all ARIA structure ensembles that we identified as reliable were actually accurate, using either unrefined or refined peak lists.

### Re-calculation of problematic targets with manually optimised parameters

As we have shown above, we identified five targets (YR313A, OR36, HR8254A, StT322 and HR5460A) as problematic for ARIA calculations using unrefined peaks lists. They can be classified in two categories: (1) proteins with more than 100 residues (YR313A 119 a.a, OR36 134 a.a. and HR5460A 160 a.a.) and (2) small proteins with unfolded tails or protruding regions without long range correlations with the globular part (StT322 63 a.a. and HR8254A 73 a.a.). In all cases, we tried to manually optimise ARIA protocol parameters to obtain converged and accurate ensembles. For the first class of problematic targets (large proteins), we managed to establish a consensus setup of parameters (Supplementary Table S4 for details). First, the total number of cooling steps for the simulated annealing (SA) protocol was increased to 60,000. In fact, it was shown that a slower cooling increased the efficiency of SA for highly ambiguous data (Fossi et al. 2005). Second, RDC restraints were introduced at a later stage of the ARIA iterative protocol (5th iteration). This insures that RDC restraints are applied only when a reasonable fold has been reached. Finally, we used a “restraint combination” approach during the first three ARIA iterations to prevent destructive effects of noise peaks in the data (Herrmann et al. 2002). The simultaneous application of these three conditions allowed us to determine accurate structures for the three larger targets YR313A, OR36 and HR5460A from unrefined peak lists when the standard protocol failed (Figs. 5, 6). For these three targets, the RMSD from the reference structure is smaller than 1.3 Å whereas it was greater than 9.0 Å when using a non-optimised protocol.

**Fig. 7:**
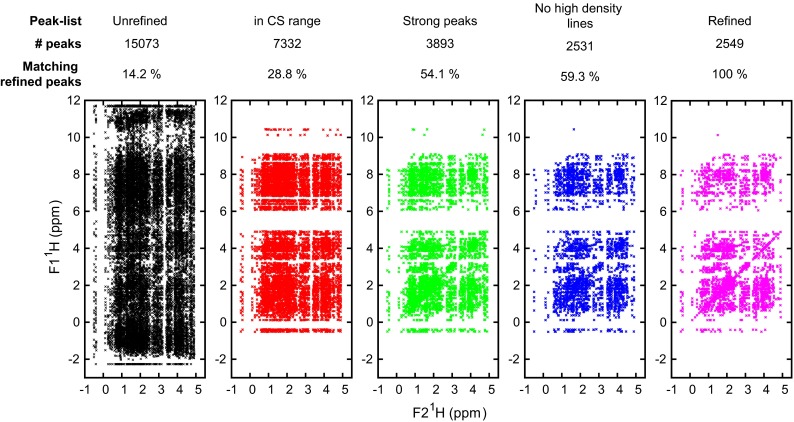
Example of peak list filtering results for the 3D ${}^{13}\hbox {C}$ NOESY peak lists of CASD–NMR target HR8254A. The cross-peak positions are projected on the ${}^1\hbox {H}$–${}^1\hbox {H}$ plane. For each peak list, the number of cross-peaks is given along with the percentage of cross-peaks having a match in the refined peak list. See “Material and methods” section for a definition of the filters applied

However, this optimised setup did not succeed for the second class of problematic targets, the smaller proteins. The structure of HR8254A (PDB 2M2E) is composed of two short $\alpha$ helices and a long and straight C-terminal $\alpha$ helix of which the last 20 a.a. are far away from the core domain (Fig. 6). Most calculations that we tried on HR8254A using unrefined peak list displayed a bent C-terminal helix. We attributed this behaviour to the large number of potential spurious cross-peaks in the unrefined data set compared to the refined one (Table 2). In fact, only 14 % of the peak present in the aliphatic region of the unrefined ${}^{13}\hbox {C}$ NOESY peak list had been conserved in the manually refined peak list (Fig. 7 and Supplementary Table S3). To circumvent this issue, we implemented two data pre-filtering procedures that discard peaks that are likely erroneous, prior to the initial NOE assignment performed by ARIA. The first filter consists of eliminating weak peaks that may be less reliable than stronger peaks in the data set. A second filter aims at detecting and discarding artifactual signal from the solvent (“high-density lines”, see “Material and methods” section for details). As a result, the successive application of the two filters on the unrefined peak list permits to enrich the number of true peaks, i.e. peaks that have been kept in the manually refined list. For instance, in the case of target HR8254A, the filtered ${}^{13}\hbox {C}$ NOESY unrefined peak list contains 2531 cross-peaks (compared to 15,073 in the raw peak list) and almost 60 % of them are also present in the manually refined peak list. Consequently, structures calculated by ARIA with the filtered peak lists for HR8254A have an accuracy of 1.52 Å, whereas an accuracy of 11.37 Å was obtained with the unfiltered peak lists. For sake of efficiency, a “network-anchoring” analysis (Herrmann et al. 2002; Bardiaux et al. 2009) was also used in the case of HR8254A only.

We tested the same filtering of unrefined peak list on the second small target, StT322. This 63 a.a. protein has a mainly $\beta$ structure, in which the first 22 residues are not structured (PDB 2LOJ) but for which chemical shifts could be assigned. From the unrefined peak list, the fold obtained by ARIA is incorrect (RMSD of 6.8 Å to the reference PDB structure). An ARIA calculation, denoted ARIA(1), was performed using pre-filtering of the unrefined peak list, longer SA cooling and “restraint combination”: consequently the RMSD of the ARIA structure to the reference PDB structure is improved up to 3.50 Å for the structured part of the protein (residues 26 to 62). Interestingly, when considering only the $\beta$-sheet region (residues 38–62), the accuracy of the ARIA ensemble is only 1.5 Å (Supplementary Figure S2). From this ensemble of StT322 structures, we inferred hydrogen-bond restraints (observed in more than 90 % of 50 best water refined conformers). A second ARIA calculation, ARIA (2), was performed on StT322, with the hydrogen-bond restraints. This yielded a slightly different fold with a different orientation of the region spanning residues 26–38, with a RMSD of 6.1 Å to the reference structure on residues 26–62. At this stage, the StT322 target appeared to be the most difficult case for ARIA since we could not find a set of parameters that would enable ARIA to obtain a highly accurate structure from unrefined NOE peak lists. It is also relevant to notice that at least two other well established approaches for NMR structure calculation also failed to determined a structure of StT322 with an accuracy smaller than 3 Å from the same data set (Zhang et al. 2014; Buchner and Güntert 2015). A similarity search of the StT322 sequence in the PDB returned another NESG target (RpT6, PDB 2JRA) which is a domain-swapped dimer. The two homologous sequences share 60 % similarity for the region corresponding to residues 38 to 63 in StT322 and the RMSD between the two structures for the same region is only 1.2 Å. We thus compared both the ARIA (1) and ARIA (2) ensembles of StT322 to the homologous dimeric structure 2JRA and it appears that the ARIA (2) structure is very similar to the monomer structure of 2JRA (Supplementary Figure S2). This leads us to speculate if the ARIA ensembles calculated on the monomeric StT322 data set are truly erroneous or if a minor dimeric form of StT322 could have been picked up in the unrefined NOE peak list, even though it seems rather unlikely when considering the careful analysis usually performed by NESG scientists in this matter (Nabuurs et al. 2006; Lee et al. 2010).

To summarise, at the exception of the StT322 target, finely optimised parameters and data pre-filtering were necessary but sufficient to obtain accurate NMR structure of target previously identified as problematic for ARIA structure calculation from raw NOE peak lists. Considering the improvement to the ARIA protocol presented in this work, we propose a set of recommended parameters for automated structure calculation with ARIA (Supplementary Table S6). We will also update the default parameters in ARIA 2.3 and make the pre-filtering procedures available for the community (aria.pasteur.fr).

## Conclusion

In the present paper, we showed that the introduction of Bayesian concepts into automated iterative structure calculations with ARIA can significantly improve the results, in particular if calculation parameters optimised for the “classical” structure calculation are appropriately modified. In particular, we introduced the log-harmonic potential together with an automated weighting procedure that we had shown to have several advantages (Nilges et al. 2008; Bernard et al. 2011) into the automated structure calculation framework.

The improved calculation set up was used “blind” on the ten CASD–NMR 2 targets, both with refined and unrefined peak lists. Structures generated by ARIA from refined NOE data sets were consistently accurate, i.e. extremely similar to the final structures determined independently by experienced scientists from the same data. In a more realistic scenario, corresponding to the use of raw NOE peak lists, ARIA managed to generate precise and accurate structures for only half of the targets, while the other half was objectively identified as unreliable. Consequently, we have developed an automated pre-filtering procedure to clean the data prior to calculation with ARIA. This allowed us, when combined with longer simulated annealing times for the larger proteins, to significantly improve the efficiency and reliability of ARIA when used with unrefined peak lists. Overall, the finely-tuned parameters for ARIA, input data filtering and validation criteria presented here are helpful for the determination and refinement of reliable and high-quality NMR structures.

**Table 1:** Protein targets from the CASD–NMR 1 data set (Rosato et al. 2012), used for the development of ARIA protocols presented here

Target name	Sequence length	No. of peak lists	No. of peaks	Residue range for RMSD	PDB entry
Vpr247	102	3	5756	2–13,21–31,35–46,57–58,68–80,92–97	2KIF
atc0905	118	3	8036	4–19,22–27,36–41,61–66,70–93,97–102	2KNR
PGR122A	73	3	3515	418–423,426–432,437–443,447–451,453–457,460–462,472–478	2KMM
HR5537A	135	2	8370	39–54,59–79,83–105,117–134	2KK1
ET109A_ox	102	3	6751	91–101,107–110,129–133,140–155,168–170,174–180,184–188	2KKY
ET109A_red	102	3	6474	91–101,107–110,114–117,146–155, 177–180,184–188	2KKX
CtR69A	63	3	1975	8–16,19–36,43–53	2KRU
CGR26A	146	3	5133	57–59,66–83,86–92,100–111,116–132,138–154,157–168	2KPT
NeR103A	105	3	4648	23–33,42–52,58–61,67–76,91–96	2KPM

For each protein, the number of residues, the number of peak lists, and the total number of peaks, as well as the residue ranges used for RMSD calculations and the corresponding PDB entry, are given

**Table 2:** Protein targets from the CASD–NMR 2 data set

Target name	Sequence length	No. of peak lists	No. of peaks (unrefined/refined)	Residue range for RMSD	PDB entry
HR6470A	69	3	4262/4216	12–58	2L9R
HR6430A	99	3	6825/6643	14–99	2LA6
HR5460A	160	3	17,250/12,015	12–158	2LAH
OR36	129	3	13,794/9459	2–128	2LCI
OR135	83	3	7749/6359	4–73	2LN3
StT322	63	4	12,437/2727	26–62	2LOJ
HR2876B	107	3	14,102/7054	12–105	2LTM
YR313A	119	3	12,303/6592	17–111	2LTL
HR8254A	73	3	19,262/3565	553–612	2M2E
HR2876C	97	3	9299/6337	17–93	2M5O

For each protein, the number of residues, the number of peak lists, and the total number of peaks (unrefined and refined), as well as the residue ranges used for RMSD calculation and corresponding PDB entry, are given

**Table 3:** van der Waals radii of hydrogen atoms for hydrogen–hydrogen interactions in the version of the PROLSQ force field used in ARIA

Hydrogen type	CNS atom type	Former radius (Å)	New radius (Å)
H aliphatic	HA	1.0	1.2
H amide	H	0.8	1.0
H charged	HC	0.8	1.0

**Table 4:** Precision (convergence) and accuracy (RMSD from the reference structure) of the CASD–NMR targets Vpr247 and atc0905 using standard or adaptive criterion for the violation tolerance determination

Target name	Potential/force field	No. of conformers per iteration	Violation tolerance	Backbone precision (Å)	Backbone accuracy (Å)
VpR247	FBHW	50	Standard	0.53 ± 0.10	1.75
VpR247	LogH	50	Standard	7.70 ± 1.19	9.44
VpR247	LogHs*	50	Standard	5.15 ± 1.37	6.30
VpR247	LogHs*	500	Standard	1.31 ± 0.49	1.41
VpR247	LogHs*	200	Manual^a^	0.73 ± 0.27	1.25
VpR247	LogHs*	50	Adaptive	0.77 ± 0.14	1.12
atc0905	FBHW	50	Standard	1.87 ± 0.39	1.52
atc0905	LogH	50	Standard	1.20 ± 0.43	1.46
atc0905	LogHs*	50	Standard	0.72 ± 0.18	1.55
atc0905	LogHs*	50	Adaptive	0.54 ± 0.15	1.34

^a^Manual determination of the optimal violation tolerance parameters *t* to achieve convergence (final values: 1000, 6, 4, 2, 2, 2, 2, 1.1, 1.1 Å)

## Electronic supplementary material

Supplementary material 1 (pdf 1188 KB)
