# Supplementary material for: Improved reliability, accuracy and quality in automated NMR structure calculation with ARIA
Source: J Biomol NMR. 2015 Apr 11;62(4):425–38. doi: 10.1007/s10858-015-9928-5 (PMC4569677; doi:10.1007/s10858-015-9928-5)
Supplement: Supplementary file 1 — Supplementary material 1 (pdf 1188 KB) [file 10858_2015_9928_MOESM1_ESM.pdf]

Supplementary Materials

for the manuscript

Improved reliability, accuracy and quality in automated NMR structure calculation with ARIA

by Fabien Mareuil, Thérèse E. Malliavin, Michael Nilges and Benjamin Bardiaux*

Unité de Bioinformatique Structurale, CNRS UMR 3528, Institut Pasteur, 25-28 rue du Dr Roux, F-75724 Paris Cedex 15, France

*To whom correspondence should be addressed. E-mail: bardiaux@pasteur.fr

Supplementary Table S1: Structure validation scores for the 10 CASD-NMR 2 targets calculated with ARIA.

Target	Peak list	Precision (Å)	% Green residues	Backbone Z-score	$\chi 1/\chi 2$ Z-score	Accuracy Cα (Å)	Accuracy Backbone	GDT-TS (%)	GDT-HA (%)	Precision[a] (Å)	Molprobity Z-score	Procheck Z-score	Accuracy[a] Cα (Å)	Accuracy[a] Backbone)	GDT-TS[a] (%)
HR2876C	refined	0.13	59.79	-0.68	-1.03	1.12	1.09	90.9	73.1	0.1	-1.23	-0.47	1.11	1.14	93
	unrefined	0.35	65.98	0.73	-0.69	0.78	0.74	97.1	81.2	0.4	-2.23	-0.18	0.79	0.80	98
HR8254A	refined	0.54	86.30	1.33	1.33	1.80	1.71	75.8	52.5	0.8	-1.52	2.66	2.13	2.06	78
	unrefined [b]	0.49	**16.44**	-6.97	-3.12	11.54	11.37	10.0	1.7	0.5	-8.19	-2.84	13.45	13.36	26
YR313A	refined	0.48	64.55	-0.42	-0.51	1.25	1.21	84.9	61.4	0.5	-1.03	-1.24	1.14	1.19	89
	unrefined[b]	**9.26**	20.00	-2.91	-4.40	9.55	9.42	14.2	3.1	8.2	-3.87	-3.73	11.80	11.77	20
HR2876B	refined	0.49	62.89	-0.15	-0.53	0.57	0.55	98.7	89.4	0.4	-1.32	-1.06	0.60	0.76	99
	unrefined	0.58	54.64	-0.96	-1.89	0.90	0.85	94.9	80.3	0.5	-4.18	-2.37	0.91	1.01	96
StT322	refined	0.41	54.10	-0.09	-0.85	1.80	1.68	83.1	63.5	2.4	-1.08	-2.66	1.84	1.81	85
	unrefined[b]	1.39	**14.75**	-1.67	-5.46	7.02	6.89	25.0	5.0	1.2	-8.85	-5.85	8.76	8.63	34
OR135	refined	0.48	64.94	2.28	-0.47	0.66	0.64	98.6	83.9	0.3	-1.18	-0.12	0.63	0.63	99
	unrefined	0.52	66.23	1.89	-1.06	0.83	0.80	95.0	79.3	0.4	-1.68	-0.41	0.74	0.75	97
OR36	refined	0.62	68.70	1.12	-1.03	0.79	0.75	95.9	81.3	0.5	-1.12	0.18	0.79	0.77	97
	unrefined[b]	**7.84**	31.01	-2.76	-3.99	9.85	9.77	17.9	2.8	6.2	-6.31	-2.78	13.84	13.90	30
HR5460A	refined	0.50	70.00	-0.05	-1.66	1.36	1.34	85.0	62.4	0.5	-2.81	0.59	1.28	1.31	89
	unrefined[b]	1.10	**10.67**	-5.80	-7.43	11.58	11.46	9.9	1.9	1.1	-40.30	-8.28	11.62	11.66	23
HR6430A	refined	0.43	68.54	0.18	-1.11	0.66	0.64	97.4	84.9	0.3	-1.13	-0.77	0.64	0.70	98
	unrefined	0.39	68.54	-0.12	-1.16	0.71	0.69	95.9	81.1	0.3	-1.61	-0.65	0.70	0.76	97
HR6470A	refined	0.38	80.00	0.84	1.64	0.59	0.56	98.4	86.2	0.3	0.20	2.19	0.58	0.59	98
	unrefined	0.52	78.33	0.72	1.34	0.47	0.44	98.9	95.2	0.3	-0.40	1.89	0.48	0.49	99

[a] Calculated on a subset of residues corresponding to the ordered residues determined by PSVS in the reference PDB structure.
[b] Considered as unsuccessful (and not submitted to evaluation) for not satisfying the criterion in **bold**

Supplementary Table S2: Structure validation scores for the 5 CASD-NMR 2 targets re-calculated with ARIA using unrefined peak lists and manually adjusted parameters

Target	Precision (Å)	% Green residues	Backbone Z-score	$\chi 1/\chi 2$ Z-score	Accuracy $C\alpha$ (Å)	Accuracy Backbone	GDT-TS (%)	Molprobity Z-score	Procheck Z-score
HR8254A	0.44	86.30	1.05	0.22	1.60	1.52	79.2	-1.91	2.54
YR313A	0.38	67.23	0.02	-1.59	1.06	1.03	90.1	-1.54	-1.01
StT322	0.69	50.79	0.75	-2.16	3.50	3.43	58.1	-1.15	-2.78
OR36	0.37	73.13	1.42	-1.09	1.03	0.99	94.3	-3.77	0.59
HR5460A	0.37	67.50	-0.74	-1.79	1.27	1.25	86.2	-2.57	0.65

Supplementary Table S3: Percentage of cross-peaks from unrefined peak lists having a match in the corresponding refined peak lists according to the type of filtering applied for 5 problematic CASD-NMR 2 targets.

Target name	NOESY peak list	Applied filters (additive)			
		None	Out of CS range	Discard weak peaks	Discard high density lines (1σ / 2σ / 3σ)
HR5460A	^{13}C-ali	36.8	53.8	64.8	73.1 / 70.9 / 67.8
HR5460A	^{13}C-aro	58.2	-	-	-
HR5460A	^{15}N	56.6	68.7	90.1	-
HR8254A	^{13}C-ali	14.2	28.8	54.1	59.3 / 56.6 / 55.2
HR8254A	^{13}C-aro	25.9	43.3	61.3	-
HR8254A	^{15}N	19.5	40.0	62.9	63.0 / 62.7 / 62.9
OR36	^{13}C-ali	36.7	57.7	77.1	80.5 / 81.5 / 80.8
OR36	^{13}C-aro	50.9	-	-	-
OR36	^{15}N	61.0	-	-	-
StT322	^{13}C-ali (h2o)	36.2	48.7	68.4	88.7 / 84.5 / 68.6
StT322	^{15}N	35.2	48.1	80.6	-
YR313A	^{13}C-ali	32.1	51.6	61.7	83.9 / 79.6 / 72.4
YR313A	^{13}C-aro	47.3	64.1	94.9	-
YR313A	^{13}N	64.3	84.9	100.0	-

Supplementary Table S4: ARIA parameters used for automated structure determination of 18 CASD-NMR targets. Chemical shift tolerances are used for initial NOE cross-peak assignment by matching cross-peak positions with the chemical shift assignments list. The number of conformers generated at every iteration and the total number of cooling steps for the simulated-annealing are also reported.

Data-set	Target name	peak lists	^{1}H tolerance (ppm)	^{13}C/^{15}N tolerance (ppm)	# conformers it0-7	# conformers it8	# cooling steps
CASD-NMR 1	NeR103A	refined	0.04	0.50	50	50	18000
	CGR26A	refined	0.04	0.50	50	50	18000
	CtR69A	refined	0.04	0.50	50	50	18000
	redET109A	refined	0.04	0.50	50	50	36000
	oxET109A	refined	0.04	0.50	50	50	36000
	atc0905	refined	0.04	0.50	50	50	18000
	HR5537A	refined	0.01	0.10	50	50	36000
	PGR122A	refined	0.02	0.20	50	50	18000
CASD-NMR 2 blind	HR6470A	refined	0.04	0.50	50	50	9000
	HR6470A	unrefined	0.04	0.50	50	50	9000
	HR6430A	refined	0.04	0.50	100	100	9000
	HR6430A	unrefined	0.04	0.50	100	100	9000
	HR5460A	refined	0.04	0.50	50	50	9000
	HR5460A	unrefined	0.01	0.10	100	100	9000
	OR36	refined	0.01	0.10	100	100	9000
	OR36	unrefined	0.01	0.10	100	100	9000
	OR135	refined	0.01	0.10	100	100	9000
	OR135	unrefined	0.01	0.10	100	100	9000
	StT322	refined	0.01	0.10	100	100	90000
	StT322	unrefined	0.01	0.10	200	200	9000
	HR2876B	refined	0.01	0.10	100	100	9000
	HR2876B	unrefined	0.01	0.10	200	200	90000
	YR313A	refined	0.01	0.10	100	100	90000
	YR313A	unrefined	0.01	0.10	100	100	9000
	HR8254A	refined	0.04	0.50	50	50	40000
	HR8254A	unrefined	0.03	0.40	100	100	60000
	HR2876C	refined	0.04	0.40	20	50	40000
	HR2876C	unrefined	0.02	0.40	60	100	30000
CASD-NMR 2 re-calculated	HR5460A	unrefined	0.02	0.40	50	100	60000
	OR36	unrefined	0.02	0.40	50	100	60000
	StT322	unrefined	0.02	0.40	50	100	60000
	YR313A	unrefined	0.02	0.40	50	100	60000
	HR8254A	unrefined	0.02	0.40	50	50	60000

Supplementary Table S5: Recommended parameters for automated NOE assignment and structure calculation with ARIA (default values for all other non-mentioned parameters).

Parameter	Recommended value
**3D NOESY peak list pre-filtering**	
^{13}C aromatic	None
^{13}C aliphatic	Discard weak peaks + discard high density lines
^{15}N	Discard weak peaks
**NOESY peak list frequency windows**	
^{1}H	0.02 ppm
^{13}C/^{15}N	0.4 ppm
**Simulated-annealing**	
Distance restraint potential	log-harmonic
Automated weighting	yes
# steps high-temperature	20000
# steps cooling 1	30000
# steps cooling 2	30000
**Force field**	
Bond and improper angles force constants	50 kcal.mol^{-1}.rad^{-2}
H vdW radii for H-H interactions	1.0/1.2 Å
**Distance restraints**	
Restraint combination 4→4	iteration 0 to 3
Violation analysis	adaptive
Violation tolerance it0	200.0
Violation tolerance it1	6.0
Violation tolerance it2	3.0
Violation tolerance it3	2.0
Violation tolerance it4	1.0
Violation tolerance it5	1.0
Violation tolerance it6	0.5
Violation tolerance it7	0.5
Violation tolerance it8	0.5
**RDC restraints**	iteration 4 to 8
**Number of conformers per iteration**	calculated/analysed
iteration 0 to 7	50/15
iteration 8	100/15
**Reliability criterion of final ensemble**	
% of green residues (CING)	>40 %
and	
Precision for ordered residues	<1.5 Å

Supplementary Table S6: Precision and accuracy for 5 CASD-NMR 2 targets calculated with ARIA using unrefined or refined peak lists with the recommended parameters given in Supplementary Table S5.

Target	peak lists	Precision Backbone (Å)	Accuracy Cα (Å)	Accuracy Backbone (Å)	GDT-TS (%)
HR2876C	unrefined	0.37	0.97	0.92	94.8
	refined	0.33	0.81	0.79	97.1
HR2876B	unrefined	0.47	0.60	0.59	98.7
	refined	0.48	0.56	0.56	98.9
OR135	unrefined	0.28	0.66	0.64	97.9
	refined	0.20	0.52	0.51	99.3
HR6430A	unrefined	0.26	0.73	0.72	95.9
	refined	0.27	0.79	0.77	95.1
HR6470A	unrefined	0.27	0.52	0.50	99.5
	refined	0.28	0.56	0.55	98.9

Supplementary Figure S1: Pairwise scatterplot (top-half) and correlation coefficients (bottom-half) between average validation scores of structures calculated by ARIA on the 10 CASD-NMR 2 targets. Structures re-calculated using unrefined peak list and manually optimised parameters are included. Successful calculations are marked in green, while calculations that failed to yield accurate structures are in red.

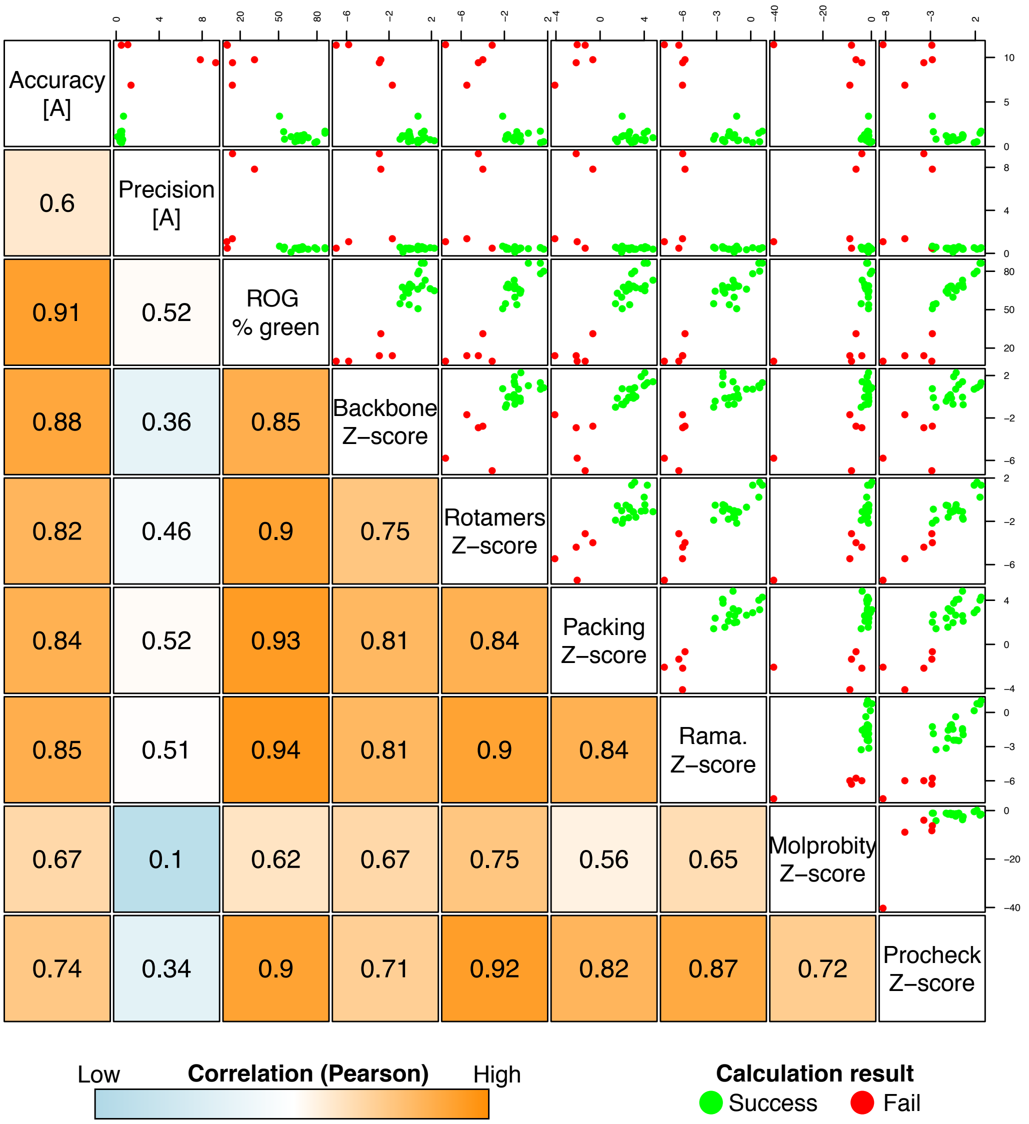

Supplementary Figure S2: Structure comparison of monomeric StT322 (PDB 2loj) and dimeric RpT6 (PDB 2jra) NESG targets. The two structures are highly similar for the region spanning residues 38 to 62 and corresponds to strands β2, β3, and β4 in StT322. The region corresponding to strand β1 and helix α1 in StT322 is swapped in RpT6. (a) Superposition of the 2loj (green) and 2jra (red/blue) PDB NMR structures. For 2jra, one monomer is coloured in blue and the other in red. (b) Superposition of the StT322 ARIA (1) ensemble (pink) on the 2loj (green) structures. ARIA (1) ensemble was calculated using filtered unrefined NOE peaks. (c) Superposition of the StT322 ARIA (2) ensemble (yellow) on one monomer of the 2jra (blue) structure. ARIA (2) ensemble of StT322 was calculated like ARIA (1) ensemble but with additional hydrogen-bond restraints inferred from the ARIA (1) ensemble. (a-c) RMSD between the superimposed structures is given for residues 26 to 62 and 38 to 62. (d) Sequence alignment and secondary structures of the StT322 (2loj) and RpT6 (2jra) targets. (e) RMSD between StT322 ARIA ensembles and the 2jra/2loj PDB structures that are not shown in (b-c).

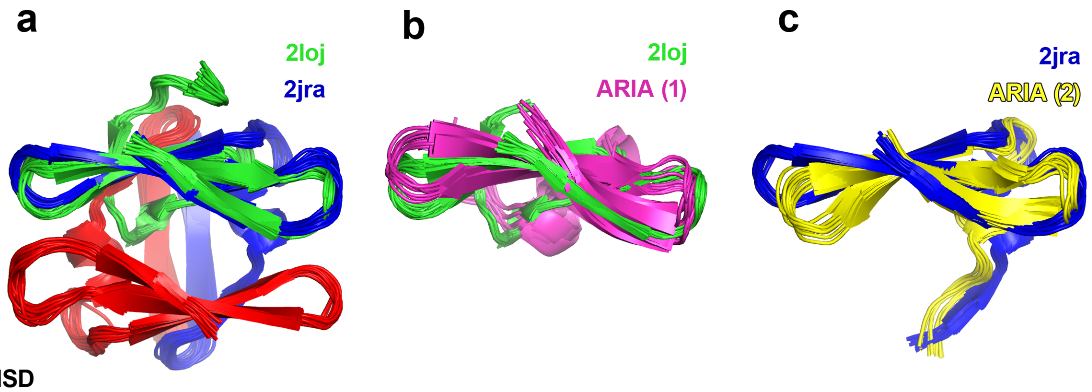

RMSD	a	b	c
26-62	6.69 Å	3.50 Å	2.26 Å
38-62	1.20 Å	1.50 Å	2.28 Å

d

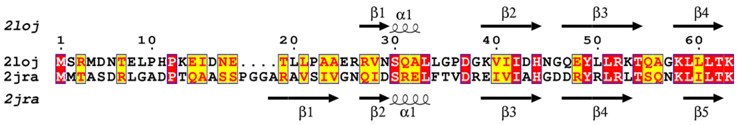

e

RMSD	ARIA (1) / 2jra	ARIA (2) / 2loj
26-62	4.81 Å	6.06 Å
38-62	1.76 Å	1.91 Å
